# PEG3 inhibits mitophagy of papillary thyroid carcinoma through ISG15/VCP axis

**DOI:** 10.1007/s00018-026-06368-x

**Published:** 2026-08-08

**Authors:** Bing Wang, Jing Yao, Zheng Wan, Xin Miao, Zelong Yang, Wen Tian, Chen Li

**Affiliations:** Senior Department of General Surgery, the First Medical Center of Chinese PLA General Hospital, 28 Fuxing Road, Haidian District, Beijing, 100853 China

**Keywords:** PTC, Gene transcription, Mitophagy, Protein interaction, Protein stability

## Abstract

Interferon-stimulated gene 15 (ISG15) is an essential biomarker in several cancers. However, the molecular mechanism of ISG15 regulating thyroid carcinoma (THCA) has rarely been studied. In our study, papillary thyroid carcinoma cell lines of TPC-1 and IHH-4, clinical samples of THCA, and nude mice were employed. The expression levels of genes were tested by real-time quantitative PCR and western blotting analyses. Cell activity was assessed using cell counting kit-8 assay and colony formation assays. Flow cytometry measured the ROS level and mitochondrial membrane potential. To induce mitophagy, cells were treated with 10µM CCCP (Carbonyl Cyanide m-Chlorophenylhydrazone) for 6 h. The level of mitophagy was assessed by transmission electron microscopy, LC3B-TOMM20 co-localization, mitochondrial Parkin level, p-S65-Ub and related protein expression. Co-immunoprecipitation assays determined the binding relationship between ISG15 and VCP. Chromatin immunoprecipitation, electrophoretic mobility shift and dual-luciferase reporter assay measured the targeting of PEG3 on the ISG15 promoter. Our results showed that ISG15 was highly expressed in THCA. ISG15 overexpression promoted cell proliferation and mitophagy. Mechanistically, ISG15 bound to VCP and promoted its stability. VCP integrated with ISG15 to promote cell viability, and mitophagy. Further experiments revealed that PEG3, which acted as a transcriptional inhibitor of ISG15, exerted an effect opposing that of ISG15. The role of ISG15 and PEG3 were also confirmed in xenograft mice model. In conclusion, our present study explored the molecular mechanism of ISG15 on THCA and found that PEG3 transcriptionally regulated ISG15 and promoted the proliferation and mitophagy of papillary thyroid carcinoma cells through VCP. Our present study revealed a novel mechanism of ISG15 on THCA, which might be a promising therapeutic target for THCA.

**Supplementary Information:**

The online version contains supplementary material available at https://doi.org/10.1007/s00018-026-06368-x.

## Introduction

Thyroid carcinoma (THCA) is the most prevalent endocrine malignancy globally, and China accounts for more than half of this burden [1]. THCA is classified into undifferentiated and differentiated subtypes. The differentiated subtype can be further categorized as papillary thyroid carcinoma (PTC), follicular thyroid carcinoma (FTC), or Hürthle cell carcinoma (HCC) [2]. Oncogenic mutations, such as those in BRAFV600E, RAS, and RET, are frequently observed in thyroid cancer and hold potential as diagnostic biomarkers. Notably, the BRAFV600E mutation is present in over 50% of THCA patients [3]. PTC represents the most common type of THCA. It usually occurs in young women, and its morbidity has increased recently [4]. The main treatment for PTC is surgical resection and subsequent radioactive iodine therapies. Usually, PTC patients have a good prognosis, especially when diagnosed early and treated appropriately [5]. However, tumor recurrence and metastasis still bother patients.

Mitophagy is a mechanism to remove damaged or dysfunctional mitochondria through the autophagic process to maintain cell energy metabolism and reduce oxidative stress [6], which is considered to involve in tumor recurrence and metastasis in several cancers [7–9]. Mitophagy is a double-edged sword. Appropriate mitophagy can digest damaged mitochondria, reduce the accumulation of reactive oxygen species (ROS), reduce cellular oxidative stress levels, and enhance cells’ adaptability to the environment [10]. However, if mitophagy occurs excessively, it will also damage the normal function of mitochondria and cause cell death [11]. Therefore, the effect of mitophagy on cancer cells will also vary depending on the environment and stress conditions.

Interferon-stimulated gene 15 (ISG15) is a ubiquitin-like protein induced by interferon, which can be connected to the receptor protein (ISGylation) through a mechanism similar to ubiquitination, thereby affecting the function of the receptor protein [12]. ISG15 has been reported to promote mitophagy. ISG15 can maintain the function of an essential mitophagy protein, Parkin, by mediating its ISGylation [13]. In pancreatic cancer stem cells, inhibition of ISG15 expression leads to accumulated damaged mitochondria, reduced oxidative phosphorylation, and inhibition of mitophagy [14]. It has been reported that ISG15 is highly expressed in PTC and associated with tumor growth and metastasis [15]. In addition, ISG15 is also found to promote cancer cell stemness, development, and doxorubicin resistance in anaplastic THCA [16]. However, the specific functions and mechanisms of ISG15 in THCA have not yet been explored.

Valosin-containing protein (VCP)/p97 is an ATPase that participates in mitochondrial outer membrane degradation and mitophagy [17]. It is a protein required for Parkin-mediated mitophagy [18]. The accumulation of VCP in mitochondria can cause excessive mitophagy [17]. When VCP is inactivated, mitochondrial membrane potential is lost, ROS increases, and mitochondrial fragments increase in cells [19]. Using BioGRID database, it is found that VCP may be a binding protein of ISG15, which means that ISG15 may mediate the ISGlytion of VCP, thereby regulating the function of VCP and mitophagy. However, this has not been verified in PTC.

Decorin-evoked paternally expressed gene 3 (PEG3) is a paternally expressed imprinted gene; that is, only the genome from the father will be expressed, while the genome from the mother will be silenced. PEG3 has a DNA binding domain and a protein binding domain. The protein binding domain allows PEG3 to bind to different proteins and co-regulate gene expression [20]. For example, by binding to the co-repressor KAP1, PEG3 can inhibit the transcription of multiple genes [20], while binding to STAT3 will promote the transcriptional activity of STAT3 [21]. In tumors, PEG3 has been reported as a tumor suppressor gene [22–24], involved in p53-mediated cell apoptosis [25], and inhibiting the wnt pathway [26]. However, this gene has not been studied in THCA. JASPAR database shows that there is a sequence in ISG15 promoter that is highly similar to the PEG3 binding motif, indicating that PEG3 may regulate the transcription of ISG15. Therefore, we propose that PEG3 may be a transcriptional factor of ISG15.

In this study, in vivo and in vitro experiments were performed to verify our hypothesis. Firstly, we assessed the function of ISG15, VCP, and PEG3 in regulating cell activity, mitophagy, migration, and invasion in THCA. Secondly, we explored the regulating relationship between ISG15 and VCP, as well as PEG3 and ISG15. Through this study, we aim at exploring the function the mechanism of ISG15 in regulating THCA in depth and providing a promising target for THCA.

## Materials and methods

### Clinical sample collection

Cancer tissues and adjacent non-cancerous tissues were collected from 30 patients with THCA who underwent surgery at our hospital. All patients signed the informed consent form. The procedures performed in this study were approved by the Ethics Committee of Chinese PLA General Hospital (S2025-362-01).

### Immunohistochemical (IHC) staining

The tissues collected from THCA paracancerous and cancer were pre-cooled with heparinized physiological saline and fixed by 4% paraformaldehyde, paraffin-embedded, and sectioned at 4-µm coronally. Then, they were incubated with primary antibodies against ISG15 (DF6316, Affinity Biosciences) at 4 ℃ for 12 h. The samples were incubated with the HRP-labeled goat anti-rabbit IgG secondary antibody at room temperature for 40 min, and observed using a light microscope.

### Cell culture and intervention

Papillary thyroid carcinoma cell lines of TPC-1 and IHH-4 were obtained from ATCC (Manassas, VA, USA), and cultured in DMEM and RPMI1640, respectively. The cells were cultured in media supplemented with 100 U/mL penicillin-streptomycin (Yeasen Biotech, China) and 10% fetal bovine serum (Biological Industries, USA) in a 5% CO_2_ incubator at 37℃.

After that, the TPC-1 and IHH-4 cells were transfected with the plasmids of vector (Vector group), overexpressed ISG15 plasmid (ISG15 group), short hairpin (sh) negative control (NC, sh-NC group), sh-ISG15 plasmid (sh-ISG15 group), overexpressed VCP and sh-NC (VCP + sh-NC group), overexpressed VCP and sh-ISG15 (VCP + sh-ISG15 group), sh-PEG3 plasmid (sh-PEG3 group), and sh-PEG3 co-transfected with sh-ISG15 (sh-PEG3 + sh-ISG15 group) using Lipofectamine 3000 (Thermo Fisher Scientific Inc., Waltham, MA, USA) at 80%–90% confluent.

### Xenograft tumor model establishment and treatments

At 5–6 weeks old, seventy male BALB/C nude mice were obtained from the Animal Center of the Chinese Academy of Sciences (Shanghai, China). The mice were kept under 12 h dark/light cycle and were available for food and water. For the xenograft experiments, TPC-1 and IHH-4 cells were injected into the armpit of animals. The tumor volume was measured every seven days. About 35 days later, the animals were anesthetized and sacrificed. Tumor volume was calculated as follows: Tumor volume=length× (width)^2^/2. The tumor weight was also measured. The xenograft tumor experiment was approved by the Ethical Committee of Chinese PLA General Hospital (S2025-362-01).

### Real-time quantitative PCR (qPCR)

The ISG15, PEG3, and VCP expression levels were evaluated in transfections. The primer of ISG15, PEG3, and VCP were designed and synthesized by Sangon (Shanghai, China), which were showed in Table 1. The qPCR experimented using HiScript II One Step qRT-PCR SYBR Green Kit (cat:#Q221-01; Vazyme Biotech Co. Ltd., Nanjing, China). Then, the ABI 7900 system (Foster City, CA, USA) processed the productions. Relative expression was calculated using the 2^–ΔΔCt^ method, referring to β-actin.

**Table 1:** Primer pairs used for quantitative RT-PCR analysis

Gene ID	Sequence (5’- 3’)
H-GAPDH-F	TGTTGCCATCAATGACCCCTT
H-GAPDH-R	CTCCACGACGTACTCAGCG
H-PEG3-F	AACATGGACTCAGGTTCCCC
H-PEG3-R	AGCAAAGGAGCACAGGTAGT
H-ISG15-F	GGTGGACAAATGCGACGAAC
H-ISG15-R	TCGAAGGTCAGCCAGAACAG
H-VCP-F	GCCGCGCAGGTTCAAAAG
H-VCP-R	AACCGATTGGGACGGTTCTT

*F* forward primer, *R* reverse primer

### Western blotting (WB) analysis

The protein in cells and tissues was obtained through RIPA lysis buffer (#R0278, Sigma). Standard sodium dodecyl sulfate-polyacrylamide gel electrophoresis (SDS-PAGE) was performed. The PVDF membrane was washed and blocked using 5% skimmed milk powder at 4 ℃ overnight. Then, the samples were incubated for 1 h with the primary antibodies against ISG15 (DF6316, Affinity Biosciences), PEG3 (LS‑C808721, LSBio), VCP (ab109240, Abcam), LC3 (ab51520, Abcam), Parkin (GTX31927, GeneTex), PINK1 (ab232374, Abcam), p-S65-Ub (ab309155, Abcam), VDAC1 (ab186321, Abcam), and β-actin (ab8227, Abcam). The samples were then incubated with a horseradish peroxidase (HRP) secondary antibody-labeled goat anti-rabbit IgG. The PVDF membrane was then incubated with electrogenerated chemiluminescence (ECL) solution (ECL808-25, Biomiga, San Diego, CA, USA) for 1 min and exposed to X-ray film.

### Cell counting kit-8 (CCK-8) assay

Cell activity was measured by the CCK-8 assay (Solarbio, Beijing, China). In brief, TPC-1 and IHH-4 cells were seeded, cultured at 37℃, treated with CCK-8 solution at 0 h, 24 h, 48 h, and 72 h, and incubated for another 2 h. Finally, the cell viability was measured at 450 nm absorbance (OD450) under a microplate reader.

### Colony formation

The cell proliferation ability was determined by counting the colony formation numbers. Cells were trypsinized, plated into 6-well plates, and cultured. After 7 days, the cells were fixed in 4% paraformaldehyde, stained with 0.1% crystal violet (Sigma-Aldrich; Merck KGaA), and observed.

### ROS measurement

Total ROS was measured using a 2’,7’-dichlorofluorescein diacetate (DCFH-DA) kit (Beyotime, Shanghai, China). Approximately 5 × 10^5^ cells/well were seeded into six-well plates and treated with 10 µmol/L DCFH-DA diluted with serum-free medium for 20 min at 37 ℃. Then, the cells were washed with a serum-free medium, and ROS levels in cells were analyzed by flow cytometry.

### Transmission electron microscopy (TEM)

To induce mitophagy, cells were treated with 10µM CCCP (Carbonyl Cyanide m-Chlorophenylhydrazone) for 6 h. A TEM experiment was performed to observe the mitophagy. The cells were initially fixed with 4% glutaraldehyde and 1% citric acid. Subsequently, the samples underwent dehydration in ethanol, embedding in Eponate 12 epoxy resin, and semi-thin sectioning using the LEICA EM UC7 Ultra-Thin Slicer. The slides were double stained with uranyl acetate and lead citrate before examination under a TEM (FEI Tecnai Spirit). Finally, the mitophagy level was quantified as the percentage of mitochondria within autophagic structures in five random fields.

### LC3B-TOMM20 co-localization detection

Mitophagy was further assessed by quantifying LC3B-TOMM20 co-localization using immunofluorescence (IF). Briefly, cells were fixed with 4% paraformaldehyde, permeabilized with 0.5% Triton X-100, and blocked with 5% goat serum. Cells were then incubated overnight at 4 °C with primary antibodies: mouse anti-LC3B (ab243506, Abcam) and rabbit anti-TOMM20 (ab186735, Abcam). After washing, cells were incubated with secondary antibodies: Alexa Fluor 647-conjugated goat anti-mouse IgG and Alexa Fluor 488-conjugated goat anti-rabbit IgG for 1 h at room temperature in the dark. Following a final wash, nuclei were counterstained with DAPI. Images were acquired using a fluorescence microscope, and co-localization was assessed.

### Mitochondrial membrane potential detection

The TPC-1 and IHH-4 cells were cultured at 70–80% confluence. Then, the cells were trypsinized, added with JC-1 dye, and cultured at 37℃ for 15 min. Finally, the cells were washed and detected through flow cytometry. The JC-1 red/green fluorescence ratio was calculated to measure the member potential changes.

### Co-immunoprecipitation (Co-IP) assay

The cells were lysed, treated with a cross-linked resin, and incubated and cultured with bead conjugated with anti-ISG15 and -VCP overnight at 4 °C. We used an elution buffer in the following day to release the protein complexes bound to the bead. The enriched complexes were then used to detect the protein level by WB.

### Chromatin immunoprecipitation (ChIP)

ChIP assay was performed on PEG3 protein and the ISG15 promoter. In brief, cells were crosslinked with formaldehyde for 10 min, incubated with anti-PEG3 and anti-IgG pre-conjugated with protein G agarose beads overnight and lysed by lysis buffer. Enrichment of ISG15 promoter was calculated using qPCR.

### Electrophoretic mobility shift assay (EMSA)

Complementary oligonucleotide probes targeting the ISG15 promoter, competitive probes without 5’-biotinylated and mutant (Mut) competitive probes were synthesized. The proteins were extracted and incubated with probes. The complexes were subjected to electrophoresis, transferred to a nylon membrane, immobilized, and detected by the chemiluminescent substrate.

### Dual-luciferase reporter assay

The promoter of ISG15 was synthetized segmentedly (GenePharma, Shanghai, China) and then inserted into the pGL3-promoter vectors (Thermo Fisher Scientific). Then, the vectors were transfected into cells and Dual-Luciferase Reporter Assay System (Promega, Madison, WI, USA) was used to detect the luciferase activity. To further confirmed the binding site of PEG3 protein on ISG15 promoter, the sequence and mutated sequence were also inserted into the reporter respectively, transfected into cells, and detected the luciferase activity.

### Bioinformatic analysis

The GEPIA database (https://ualcan.path.uab.edu/) displayed the expression level of ISG15 in THCA. The 2000 bp promoter sequence upstream of ISG15 was obtained from the NCBI database and JASPAR (https://jaspar.elixir.no/) predicted the binding sites of PEG3 on the ISG15 promoter.

### Statistical analysis

Data are exhibited as mean ± standard deviation (SD). Unpaired student’s *t*-test, one-way ANOVA analyses followed with Tukey’s test, and graphs were performed in GraphPad Prism 10 (San Diego, CA, USA), depending on the groups. *P* < 0.05 was considered a significant difference.

## Results

### ISG15 was highly expressed in THCA

**Fig. 1:**
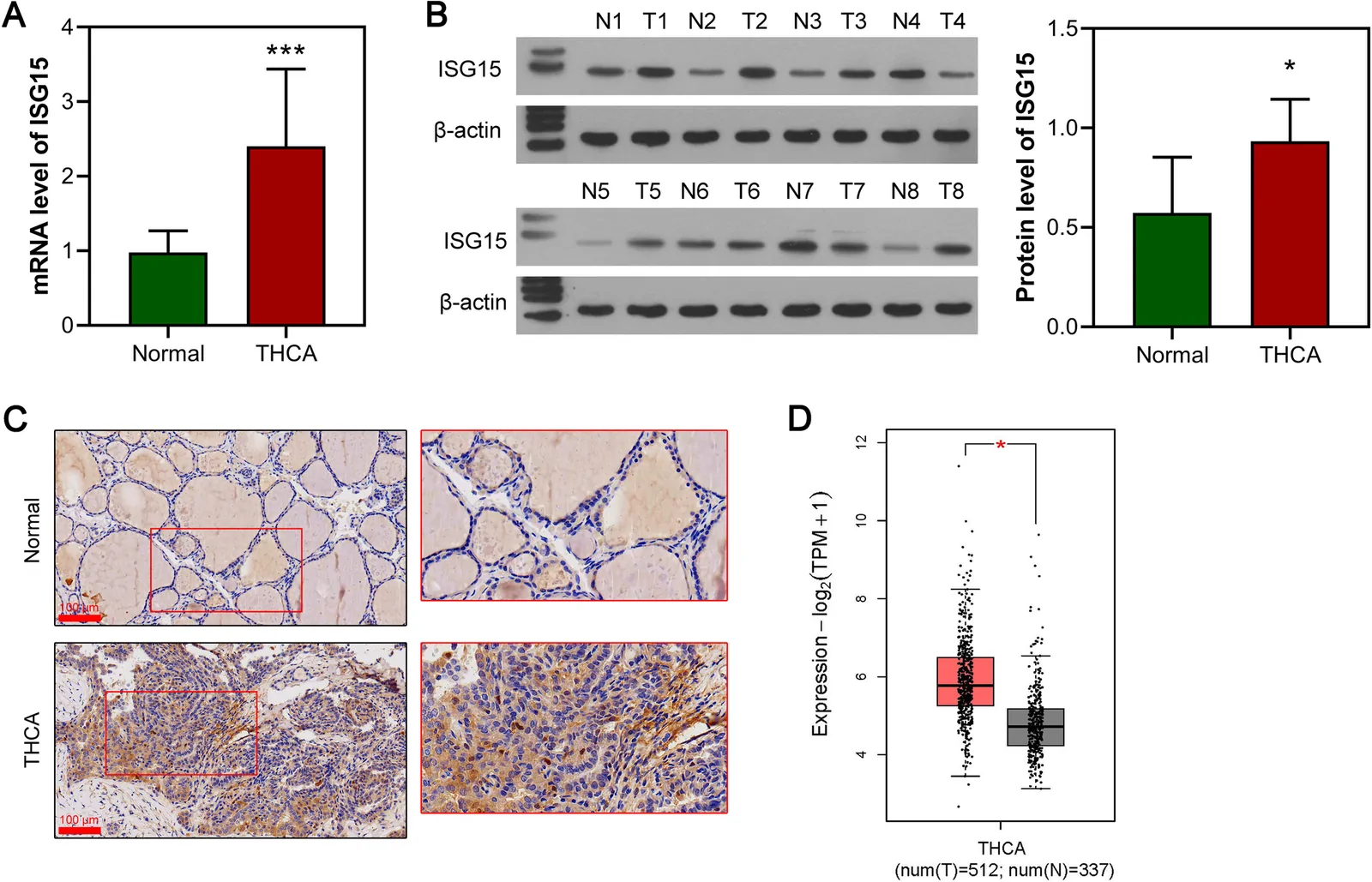
ISG15 was highly expressed in THCA. (**A**) QPCR analysis evaluated the mRNA expression levels of ISG15 in Normal and THCA tissues. (**B**) WB analysis determined the protein expression levels of ISG15, using β-actin as the reference. (**C**) IHC measured the protein expression levels of ISG15 in Normal and THCA tissues. (**D**) The expression level of ISG15 was detected in the GEPIA database. *N* = 30. Mean ± SD. *P < 0.05, ***P < 0.001

Firstly, the expression levels of ISG15 in THCA were measured in tumor and paracancerous tissues by qPCR, WB, and IHC. As detected, ISG15 was significantly highly expressed in THCA tumor tissues compared to the paracancerous tissues (*P* < 0.01, Figs. 1A-B). IHC staining showed more positive area in tumor tissues, compared with those in paracancerous tissues (Fig. 1C), demonstrating a higher expression level of ISG15 in THCA. In the GEPIA database, the expression level of ISG15 was also higher in Tumor samples than in Normal samples (Fig. 1D).

### ISG15 promoted cell viability and enhanced mitophagy

**Fig. 2:**
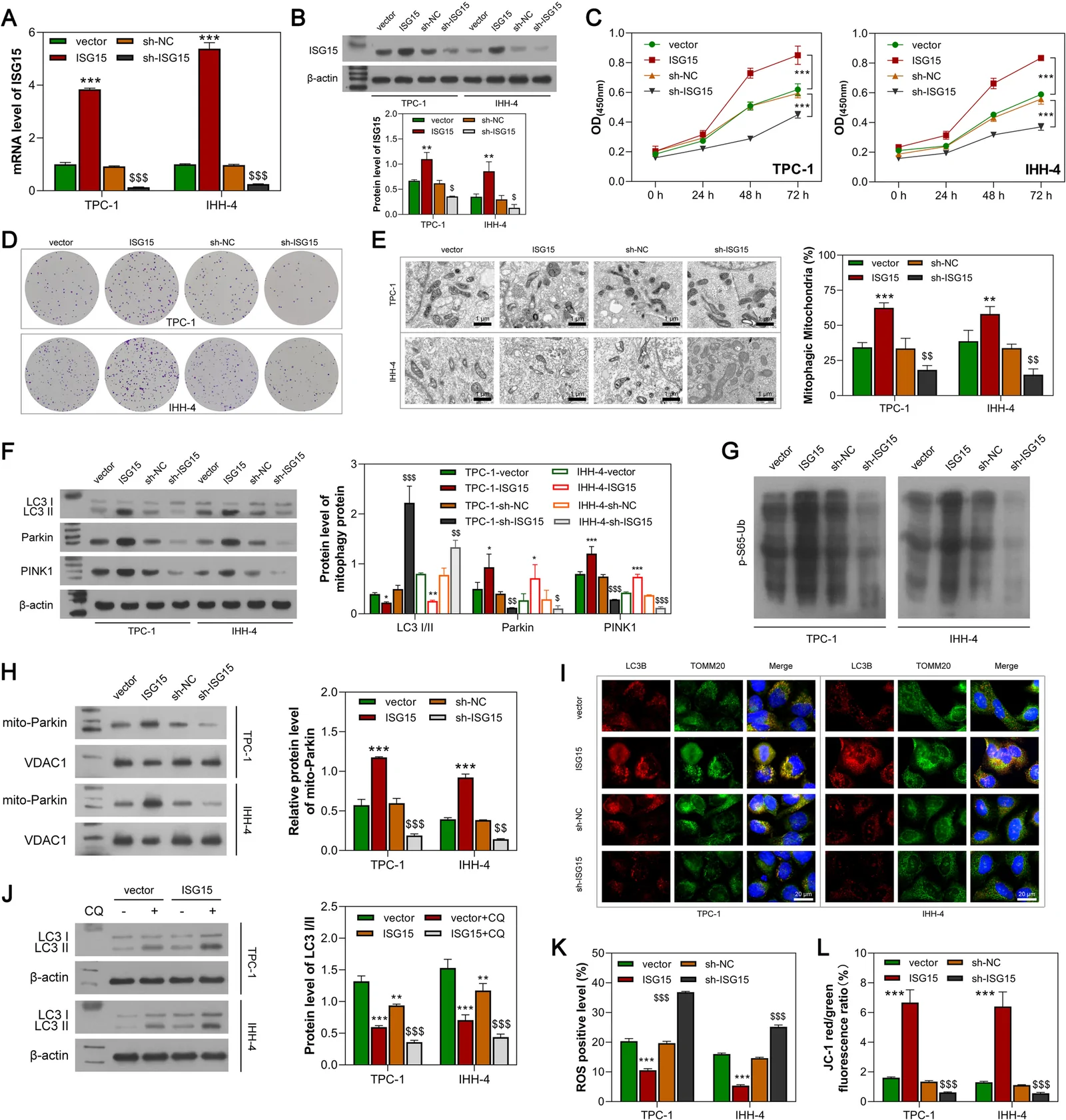
ISG15 promoted cell viability and enhanced mitophagy. (**A**) QPCR analysis evaluated the mRNA expression levels of ISG15 in transfections. (**B**) WB analysis determined the protein expression levels of ISG15 in transfections, using β-actin as the reference. (**C**) CCK-8 measured the cell activity of TPC-1 and IHH-4 cells transfected with overexpressed ISG15 and sh-ISG15. (**D**) Colony formation assay assessed the cell proliferation of TPC-1 and IHH-4 cells transfected with overexpressed ISG15 and sh-ISG15. (**E**) TEM observed the mitophagy in transfections. (**F**) WB analysis determined the protein expression levels of mitophagy biomarkers of LC3, Parkin, and PINK1 in transfections, using β-actin as the reference. (**G**-**H**) The p-S65-Ub and mitochondrial Parkin level was assessed by WB. (**I**) The co-localization of LC3B and TOMM20 was determined by IF assay. (**J**) The level of LC3 in cells treated with or without CQ was evaluated. (**K**) Flow cytometry determined the ROS level using a 2’, 7’-dichlorofluorescein diacetate (DCFH-DA) kit in transfections. (**L**) Flow cytometry assessed the mitochondrial membrane potential using JC-1 dye. *N* = 3. Mean ± SD. *P < 0.05, **P < 0.01, ***P < 0.001 vs. vector. ^$^ *P* < 0.05, ^$$^ *P* < 0.01, ^$$$^ *P* < 0.001, vs. sh-NC

Subsequently, the function of ISG15 in THCA was explored by transfecting overexpressed ISG15 and sh-ISG15 in TPC-1 and IHH-4 cells. Firstly, the expression level of ISG15 was detected in transfections by qPCR and WB analyses. As measured, the expression of ISG15 was significantly increased in the group transfected with ISG15 overexpression plasmid while decreased in the group transfected with sh-ISG15 inhibition plasmid when compared with vector and sh-NC, respectively (*P* < 0.001, Figs. 2A and B). After confirming the success of transfections, the cell activity and mitophagy level in transfections were determined. Cell viability was significantly increased in the ISG15 group and decreased in the sh-ISG15 group when compared with vector and sh-NC groups, as evaluated by CCK-8 assay (*P* < 0.001, Fig. 2C). The colony formation number in the ISG15 group was remarkably higher than that in the vector group and lower in the sh-ISG15 group than in the sh-NC group (Fig. 2D). These results revealed an enhanced cell proliferation and activity by ISG15. As detected by TEM, more autophagosomes containing mitochondria were observed in the ISG15 group and less in the sh-ISG15 group than in the vector and sh-NC groups (Fig. 2E). WB confirmed the mitophagy changes by determining LC3, Parkin, and PINK1 expression levels in transfections. Mitophagy biomarkers of LC3 II, Parkin, and PINK1 were highly expressed in the ISG15 group and lowly expressed in the sh-ISG15 group compared to vector and sh-NC groups (Fig. 2F). Moreover, p-S65-Ub levels were elevated in the ISG15 group but reduced in the sh-ISG15 group (Fig. 2G). To further assess mitophagy, we examined Parkin translocation to mitochondria and detected LC3B-TOMM20 co-localization. Western blot analysis of mitochondrial proteins revealed increased mitochondrial localization of Parkin upon ISG15 overexpression and decreased localization following ISG15 knockdown (Fig. 2H). IF analysis confirmed enhanced co-localization of LC3B and TOMM20 in the ISG15 overexpression group, while less co-localization in sh-ISG15 group (Fig. 2I). To confirm ISG15’s role in regulating mitophagy, cells were treated with lysosomal inhibitors chloroquine (CQ, 50 µM). It was found that CQ treatment increased the LC3 II level, an effect that was amplified in the ISG15 overexpression group (Fig. 2J). ROS level and mitochondrial membrane potential were detected to evaluate the mitochondrial injury. In this study, the level of ROS was significantly reduced by ISG15 and increased by sh-ISG15 (*P* < 0.001, Figs. 2K and Figure S1A). Compared with the Vector and sh-NC groups, the JC-1 red/green fluorescence ratio was significantly enhanced by overexpressed ISG15 and weakened by sh-ISG15 (*P* < 0.001, Figs. 2L and Figure S1B), revealing a promoted mitochondrial membrane potential by ISG15. The results above showed that ISG15 promoted cell viability and enhanced mitophagy.

### ISG15 bound to VCP and promotes its stability

**Fig. 3:**
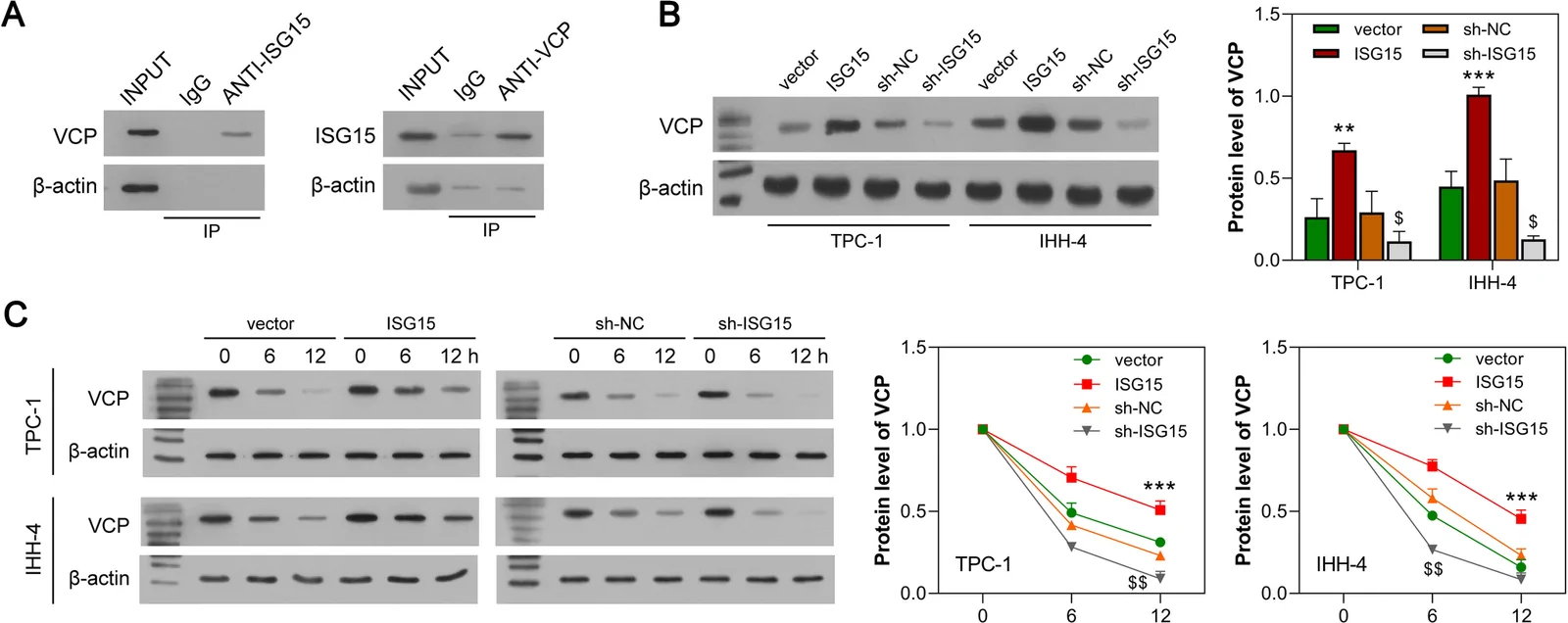
ISG15 bound to VCP and promoted its stability. (**A**) Co-IP assay was performed to investigate the binding relationship between ISG15 and VCP. (**B**) The protein expression of VCP was tested in transfections. (**C**) The protein stability of VCP was tested in transfections at 0 h, 6 h, and 12 h after treated with CHX. *N* = 3. Mean ± SD. **P < 0.01, ***P < 0.001 vs. vector. ^$^ *P* < 0.05, ^$$^ *P* < 0.01, vs. sh-NC

Using BioGRID database, it was found that VCP, one of important mitophagy-related proteins, might be a binding protein of ISG15, we then explore whether ISG15 regulated mitophagy by VCP. Co-IP assay tested the binding relationship between ISG15 and VCP. As detected, VCP protein was enriched by ISG15 antibody, and ISG15 protein was also enriched by VCP antibody (Fig. 3A), demonstrating the binding relationship between ISG15 and VCP. VCP exhibited a positive expression relationship with ISG15, with VCP highly expressed in overexpressed ISG15 group and lowly expressed in sh-ISG15 group (Fig. 3B). In addition, the VCP protein stability was tested in transfections after treatment with protein synthesis inhibitor CHX. The protein level of VCP was more stable in ISG15 group than in sh-ISG15 group after CHX treatment (Fig. 3C).

### VCP integrated with ISG15 in promoting cell viability and enhancing mitophagy

**Fig. 4:**
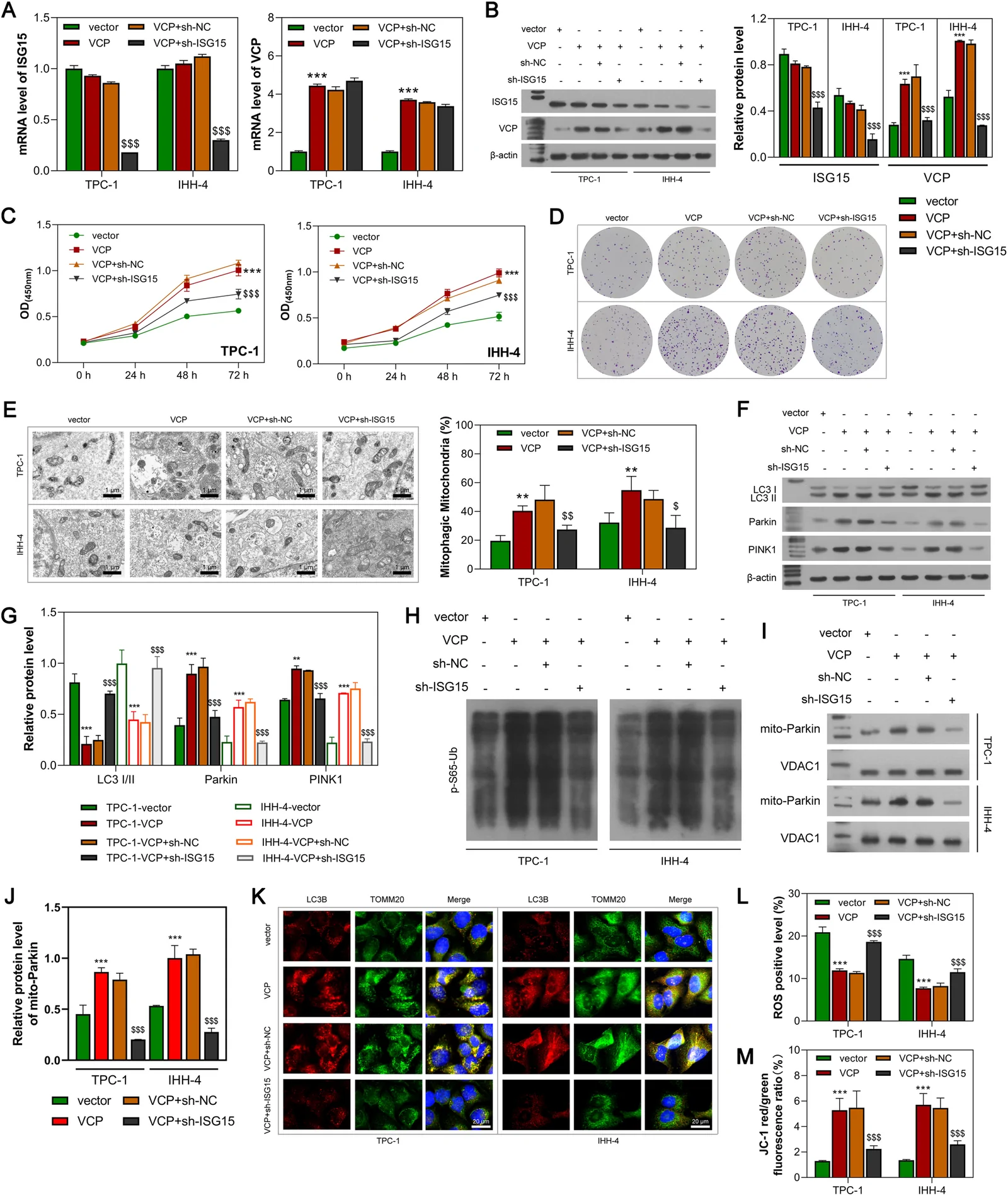
VCP integrated with ISG15 in promoting cell viability and enhancing mitophagy. (**A**) QPCR analysis evaluated the mRNA expression levels of VCP and ISG15 in transfections. (**B**) WB analysis determined the protein expression levels of VCP and ISG15 in transfections, using β-actin as the reference. (**C**) CCK-8 measured the cell activity in transfections. (**D**) Colony formation assay assessed the cell proliferation in transfections. (**E**) TEM observed the mitophagy in transfections. (**F**-**G**) WB analysis determined the protein expression levels of mitophagy biomarkers of LC3, Parkin, and PINK1 in transfections, using β-actin as the reference. (**H**-**J**) The p-S65-Ub and mitochondrial Parkin level was assessed by WB. (**K**) The co-localization of LC3B and TOMM20 was determined by IF assay. (**L**) Flow cytometry determined the ROS level using a 2’, 7’-dichlorofluorescein diacetate (DCFH-DA) kit in transfections. (**M**) Flow cytometry assessed the mitochondrial membrane potential using JC-1 dye. *N* = 3. Mean ± SD. **P < 0.01, ***P < 0.001 vs. vector. ^$^ *P* < 0.05, ^$$^ *P* < 0.01, ^$$$^ *P* < 0.001, vs. VCP + sh-NC

The function of VCP in THCA was explored through transfecting overexpressed VCP in TPC-1 and IHH-4 cells. In addition, sh-ISG15 plasmid was co-transfected with overexpressed VCP to evaluate the relationship between ISG15 and VCP. Compared with the mRNA and protein expression level of ISG15 in the VCP + sh-NC group, it is significantly decreased in the VCP + sh-ISG15 group (*P* < 0.001); VCP was significantly highly expressed in the overexpressed VCP group when compared with the vector, while its protein level was inhibited by ISG15 knockdown (*P* < 0.001, Figs. 4A and B). Additionally, we found that ISG15 and VCP did not affect each other’s mRNA levels. Compared with the cell viability in the vector group, it was significantly promoted by the overexpressed VCP group (*P* < 0.001) and then remarkably recovered by sh-ISG15 (*P* < 0.001, Fig. 4C). The colony formation numbers were significantly increased by overexpressed VCP when compared with the vector group, which were then notably reversed in the VCP + sh-ISG15 group (Fig. 4D). TEM showed that more mitophagic mitochondria were observed in the VCP group than the vector group; less in the VCP + sh-ISG15 group than in the VCP + sh-NC groups (Fig. 4E). The expression of mitophagy-related proteins of LC3 II, Parkin, and PINK1 was remarkably increased in the VCP overexpression group compared with the vector group, which notably decreased when co-transfected with sh-ISG15 (Fig. 4F and G). Overexpression of VCP increased p-S65-Ub levels, mitochondrial Parkin localization, and LC3B-TOMM20 co-localization, all of which were attenuated by ISG15 knockdown (Fig. 4H-K). The level of ROS was significantly decreased by overexpressed VCP and restored by VCP + sh-ISG15 (*P* < 0.001, Figs. 4L and Figure S1C). Compared with the Vector group, the JC-1 red/green fluorescence ratio was significantly enhanced by overexpressed VCP (*P* < 0.001, Figs. 4M and Figure S1D), revealing a promoted mitochondrial membrane potential by VCP. Taking the results above, we found that ISG15 stabilized VCP, therefore promoted cell viability and enhanced mitophagy.

### ISG15 promoted tumor progression in vivo

**Fig. 5:**
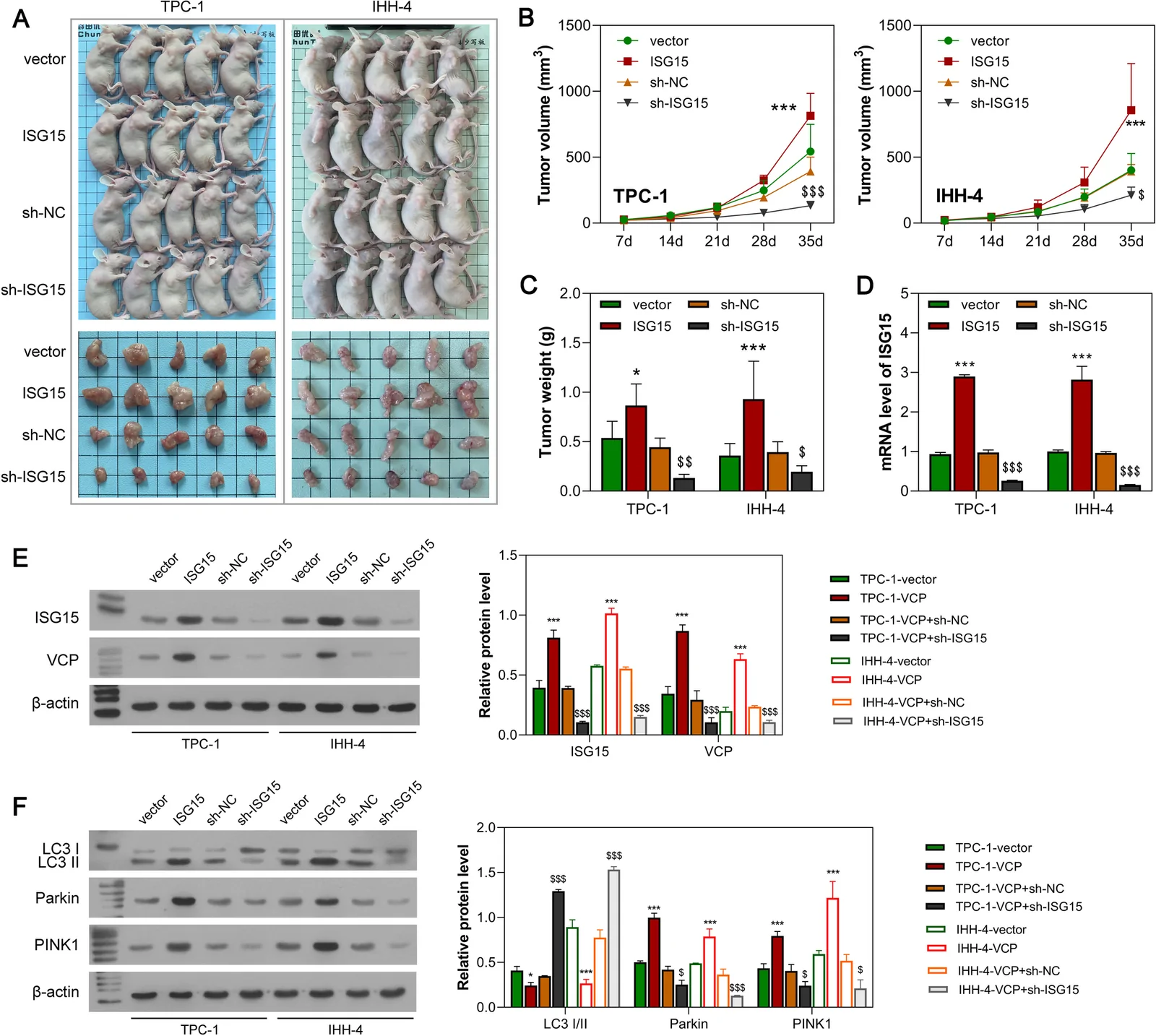
ISG15 promoted tumor progression in vivo. (**A**-**C**) showed the tumor size, tumor volume, and tumor weight of tumors. (**D**) QPCR evaluated the mRNA level of ISG15 in tumors. (**E**, **F**) WB analysis assessed the protein expression levels of ISG15, VCP, LC3, Parkin, and PINK1, using β-actin as the reference. *N* = 5. Mean ± SD.^*^P < 0.05, ^***^P < 0.001 vs. vector. ^$^ *P* < 0.05, ^$$^ *P* < 0.01, ^$$$^ *P* < 0.001, vs. sh-NC

Then xenograft model was used to confirmed the role of ISG15. In vivo, the tumor size, volume, and weight were significantly enlarged by overexpressed ISG15 and diminished by sh-ISG15 (*P* < 0.001, Figs. 5A-C). The expression level of ISG15 in tumor was measured. As detected, tumor with overexpressed ISG15 transfection exhibited higher mRNA and protein expression levels of ISG15 than those injected with vector; tumor in the sh-ISG15 group showed lower expression levels of ISG15 than those in sh-NC group (*P* < 0.001, Figs. 5D and E). The protein expression of VCP exhibited a positive trend, similar to that of ISG15 in transfections (Fig. 5E). LC3 II, Parkin, and PINK1 mitophagy biomarkers were notably highly expressed in ISG15 group and lowly expressed in sh-ISG15 group (Fig. 5F), revealing an enhanced mitophagy by ISG15. Therefore, in vivo experiment confirmed the promoting role of ISG15 in tumor growth and mitophagy.

### PEG3 was an ISG15 transcriptional inhibitor

In this study, we predicted PEG3 as a transcriptional factor of ISG15, and CHIP and EMSA assays were performed to validate the binding relationship. In the CHIP experiment, the PEG3 antibody enriched more ISG15 promoter sequences than IgG (*P* < 0.001, Figure S2A). The ISG15 promoter probe band was shifted by PEG3 protein, which was disappeared when supplemented with the competitive probe and further shifted when added PEG3 antibody (Figure S2B), revealing a binding relationship between PEG3 protein and the ISG15 promoter. Then, we explore the binding site of PEG3 on ISG15 promoter. As shown in Figure S2C, the ISG15 promoter was divided into six segmentations, and the dual luciferase reporter gene vector was constructed. PEG3 overexpression plasmid significantly reduced the fluorescence signal of WT, P1, P3, P5, and P6 dual-fluorescence plasmids (*P* < 0.001, Figure S2D) but not of P2 and P4, indicating that the binding sequence was located in P6 (−2000~−1839), in which a sequence resembled the PEG3 binding motif was found (Figure S2E). The binding site was further confirmed. PEG3 overexpression plasmid weakened the fluorescence signal of the P3 dual-fluorescence plasmid, and the inhibitory effect disappeared after mutating the binding sequence (*P* < 0.001, Figure S2E). Taken together, we concluded that PEG3 bound to the ISG15 promoter and inhibited its transcription.

### PEG3 inhibited cell viability and mitophagy by suppressing ISG15

**Fig. 6:**
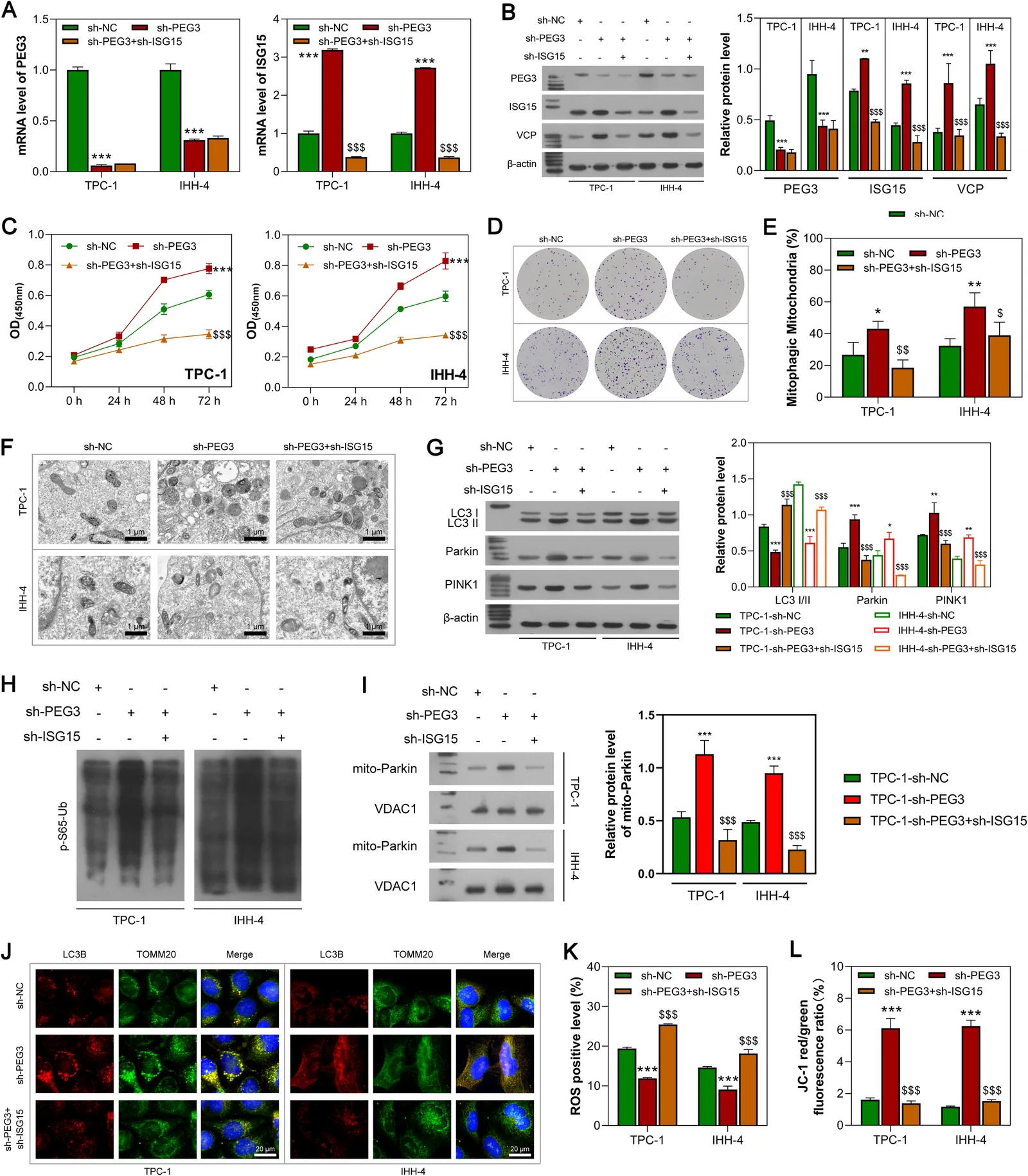
PEG3 inhibited cell viability and mitophagy by suppressing ISG15. (**A**) QPCR analysis evaluated the mRNA expression levels of PEG3 and ISG15 in transfections. (**B**) WB analysis determined the protein expression levels of VCP, PEG3, and ISG15 in transfections, using β-actin as the reference. (**C**) CCK-8 measured the cell activity in transfections in TPC-1 and IHH-4 cells. (**D**) Colony formation assay assessed the cell proliferation in transfections. (**E**-**F**) TEM observed the mitophagy in transfections. (**G**) WB analysis determined the protein expression levels of mitophagy biomarkers of LC3, Parkin, and PINK1 in transfections, using β-actin as the reference. (**H**-**I**) The p-S65-Ub and mitochondrial Parkin level was assessed by WB. (**J**) The co-localization of LC3B and TOMM20 was determined by IF assay. (**K**) Flow cytometry determined the ROS level using a 2’, 7’-dichlorofluorescein diacetate (DCFH-DA) kit in transfections. (**L**) Flow cytometry assessed the mitochondrial membrane potential using JC-1 dye. *N* = 3. Mean ± SD. *P < 0.05, **P < 0.01, ***P < 0.001 vs. sh-NC. ^$^ *P* < 0.05, ^$$^ *P* < 0.01, ^$$$^ *P* < 0.001, vs. sh-PEG3

The function of PEG3 in regulating THCA progression and mitophagy was evaluated. Transfections of sh-PEG3, sh-PEG3 + sh-ISG15, and the sh-NC were established. QPCR assessed the mRNA expression levels of PEG3 and ISG15 in sh-PEG3, sh-PEG3 + sh-ISG15, and sh-NC groups. The results showed that PEG3 was lowly expressed in sh-PEG3 and sh-PEG3 + sh-ISG15 groups compared to the sh-NC group (*P* < 0.001, Figs. 6A and B). Silencing PEG3 remarkably promoted the expression level of ISG15 (*P* < 0.001), which was then notably inhibited when co-transfected with sh-ISG15 (*P* < 0.001; Figs. 6A and B). As for the protein expression level of VCP showed a consistent regulation trend with that of ISG15 (Fig. 6B). Subsequently, the role of PEG3 in regulating cell activity was assessed. As detected, silencing PEG3 significantly increased the cell viability and colony formation number compared with sh-NC, which were all recovered when co-transfected with sh-ISG15 (*P* < 0.001, Figs. 6C and D). More mitochondria in autophagosome were observed in sh-PEG3 groups, and less were observed in the sh-PEG3 + sh-ISG15 group (Fig. 6E and F), revealing an enhanced mitophagy ability by sh-PEG3. Moreover, the sh-PEG3 group remarkably increased the expression of mitophagy-related proteins of LC3 II, Parkin, and PINK1 compared with the sh-NC group, which notably were decreased when co-transfected with sh-ISG15 (Fig. 6G). The increased p-S65-Ub level, mitochondrial Parkin localization, and LC3B-TOMM20 co-localization by sh-PEG3 were also reversed by sh-ISG15 (Fig. 6H-J). The level of ROS was significantly decreased by sh-PEG3 and restored by sh-PEG3 + sh-ISG15 (*P* < 0.001, Figs. 6K and Figure S1E). Compared with the sh-NC group, the JC-1 red/green fluorescence ratio was significantly enhanced by sh-PEG3 (*P* < 0.001, Figs. 6L and Figure S1F) and weakened by sh-PEG3 + sh-ISG15, revealing a promoted mitochondrial membrane potential by sh-PEG3. We further examined the effects of PEG3 overexpression and observed that it significantly reduced both cell viability and mitophagy levels (Figure S3). Taken the results above, we found that PEG3 inhibited cell viability and mitophagy by regulating ISG15.

### Silencing PEG3 promoted tumor progression in vivo

**Fig. 7:**
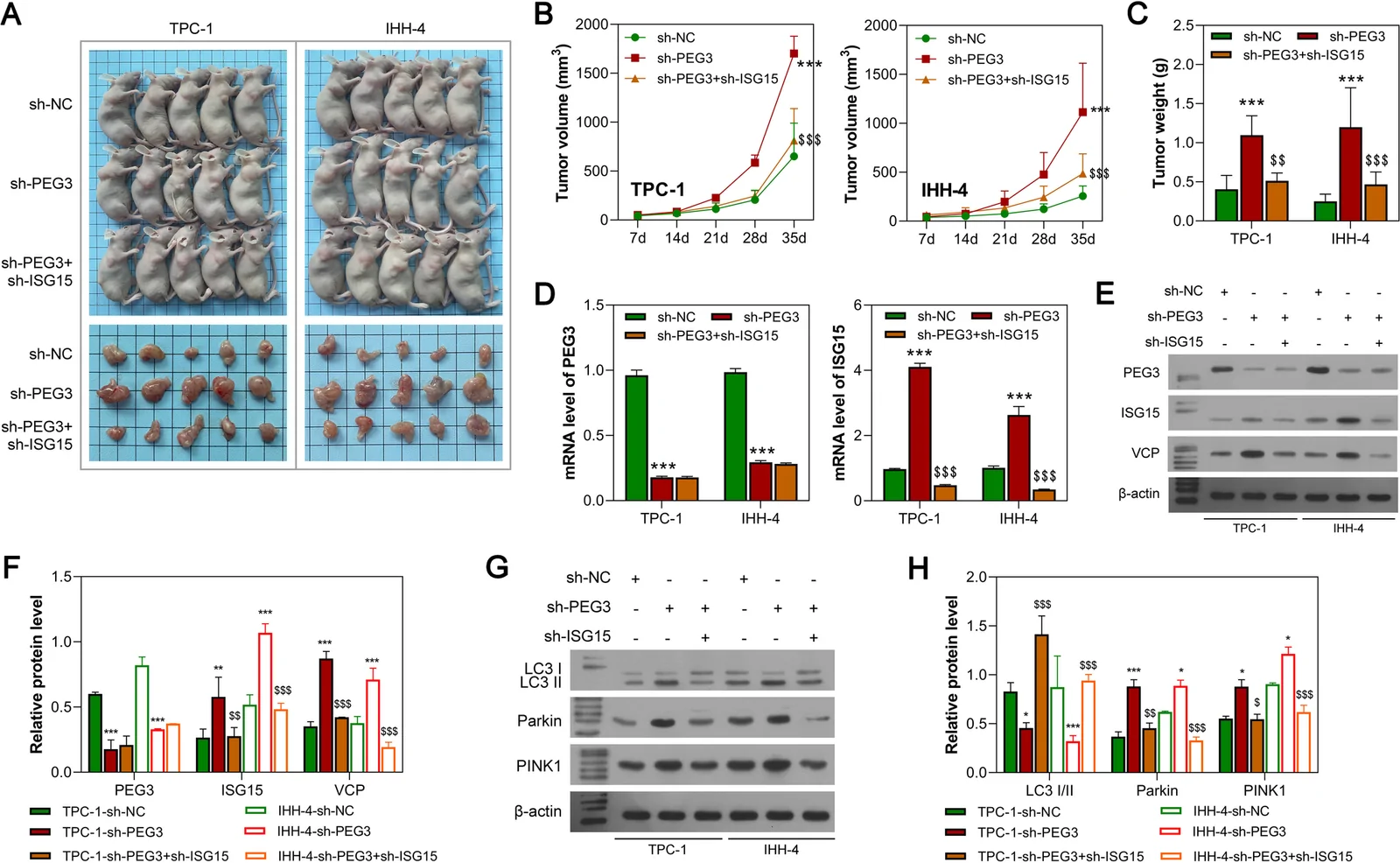
Silencing PEG3 promoted tumor growth in vivo. (**A**-**C**) showed the tumor size, tumor volume, and tumor weight of tumors. (**D**) QPCR evaluated the mRNA level of PEG3 and ISG15 in groups. (**E**-**H**) WB analysis assessed the protein expression levels of PEG3, ISG15, VCP, LC3, Parkin, and PINK1, using β-actin as the reference. *N* = 5. Mean ± SD. ^***^P < 0.001 vs. sh-NC. ^$$$^ *P* < 0.001, vs. sh-PEG3

Finally, xenograft experiments were conducted to further verify the function of PEG3 in THCA. As measured, the silencing PEG3 significantly increased the tumor size, volume, and weight compared with sh-NC (*P* < 0.01, *P* < 0.001, Figs. 7A-C). Additionally, animals injected with cells co-transfected with sh-ISG15 plasmid showed smaller tumor size, volume, and weight than sh-PEG3 (*P* < 0.001). Compared with the mRNA expression levels of PEG3 in the sh-NC group, it was significantly decreased in the sh-PEG3 and sh-PEG3 + sh-ISG15 groups (*P* < 0.001, Fig. 7D). ISG15 was notably increased after being transfected with sh-PEG3 and then reduced when co-transfected with sh-ISG15 (*P* < 0.001, Fig. 7D). WB analysis determined the PEG3, ISG15, and VCP expression levels in groups. The protein expression levels of PEG3 and ISG15 were consistent with mRNA levels (Fig. 7E and F). VCP exhibited a co-expression trend with that of ISG15 in these groups. We measured the mitophagy biomarkers in these groups. We found that the sh-PEG3 group remarkably increased LC3 II, Parkin, and PINK1 compared with the sh-NC group, which notably decreased when co-transfected with sh-ISG15, consistent with the result in vitro (Fig. 7G and H). The xenograft experiment confirmed that silencing PEG3 promoted tumor growth and mitophagy in vivo.

## Discussion

In this study, we verified the hypothesis that ISG15 regulated cell activity and mitophagy in THCA. In addition, we further explored the molecular mechanism of how ISG15 regulated THCA progression. Through this study, we found that (1) ISG15 was highly expressed in THCA and promoted tumor growth; (2) ISG15 regulated mitophagy through binding to VCP; (3) ISG15 was transcriptionally inhibited by PEG3. We concluded that PEG3 transcriptionally regulated ISG15 and promoted the proliferation and mitophagy of papillary thyroid carcinoma cells through VCP.

In this study, we found that ISG15 was highly expressed in THCA. Not only in THCA, ISG15 was reported to be an essential biomarker in various cancers [27–29]. In cervical cancer, the authors demonstrate that ISG15 is upregulated and positively associated with its development, which is considered as an oncogene in cervical cancer [30]. Yuan et al. [29]. summarize the function of ISG15/ISGylation in cancer and demonstrate that ISG15 is involved in cancer occurrence, progression, DNA repair, immune regulation, exosome secretion, and autophagy. The constitutive elevation of the ISG15 pathway leads to defects in mitophagy and increases oxidative stress in A-T cells [31]. The association between ISG15 and autophagy, especially mitophagy, has been reported in several diseases [32–35]. For example, Alcalá et al. [36]. analyze the role of ISG15 and ISGylation in regulating pancreatic cancer stem cell mitophagy, and the authors demonstrate that ISG15 is related to mitochondria-related pathways. In addition, mitophagy is impaired in the absence of ISG15, which is consistent with our present study. ISG15 and ISGylation are essential regulators of mitochondrial processes, including mitophagy, mitochondrial respiration, mitochondrial dynamics and glutamine metabolism [14, 37, 38]. Given that ISG15 is a multifunctional protein with pleiotropic effects, it may also regulate other mitochondrial functions in thyroid cancer, which merits further investigation.

VCP is one of the members of the AAA family, which is abundantly in eukaryotic cells and is associated with proteins with various functions, including transcriptional regulation, programmed cell death, membrane fusion, and ubiquitin/proteasome-dependent protein degradation [39, 40]. Clinical studies have identified the correlation between VCP and cancers, including breast cancer [41], colorectal cancer [42], lung cancer [43], and pancreatic cancer [44]. However, the function of VCP on THCA has never been reported. Our present study revealed that VCP promoted cell viability and enhanced mitophagy in THCA for the first time. In addition, we also confirmed that ISG15 bound to and stabilized VCP. The function of VCP on THCA could be recovered by silencing ISG15. We found that VCP and ISG15 integrated in regulating cell proliferation and mitophagy of THCA.

PEG3 is also reported to be an essential biomarker in cancers. For example, PEG3 is considered a promising treatment target for colon adenocarcinoma, together with BTG2 and PPP1CA [45]. Epigenetic reactivation of PEG3 by EZH2 inhibitors suppresses renal clear cell carcinoma progress [46]. No studies of PEG3 on THCA have been reported before. In our present study, we investigated the function of PEG3 on THCA and explored the transcriptional role of PEG3 on ISG15. We demonstrated that PEG3 was a transcriptional inhibitor of ISG15. We also found that PEG3 inhibited cell viability and mitophagy in both in vitro and in vivo experiments by regulating ISG15. Although there are few researches exploring the role of PEG3 in mitophagy, PEG3 has been reported to take part in autophagy. PEG3 is an upstream transcription factor EB (TFEB) regulator in endothelial cell autophagy [47]. Decorin-inducible PEG3 evokes beclin 1-mediated autophagy and thrombospondin 1-mediated angiostasis [48]. Our study revealed the role of PEG3 in mitophagy for the first time in THCA.

In summary, our present study explored the molecular mechanism of ISG15 on THCA. We found that PEG3 transcriptionally regulated ISG15 and promoted proliferation and mitophagy of papillary thyroid carcinoma cells through VCP. Our present study revealed a novel mechanism of ISG15 on THCA, which might be a promising therapeutic target for THCA.

## Supplementary information

Below is the link to the electronic supplementary material.

Supplementary Material 1 (JPG 2.16 MB)

Supplementary Material 2 (JPG 1.03 MB)

Supplementary Material 3 (JPG 1.68 MB)

Supplementary Material 4 (DOCX 13.9 KB)

## Data Availability

The data that support the findings of this study are available from the corresponding author upon reasonable request.
