# Supplementary material for: PEG3 inhibits mitophagy of papillary thyroid carcinoma through ISG15/VCP axis
Source: Cell Mol Life Sci. 2026 Aug 8;83(1):407. doi: 10.1007/s00018-026-06368-x (PMC13737257; doi:10.1007/s00018-026-06368-x)
Supplement: Supplementary file 1 [file 18_2026_6368_MOESM4_ESM.docx]

**Supplementary files**

**Figure S1. The representative images of flow cytometry detecting cellular ROS level and mitochondrial membrane potential.**

**Figure S2.** **PEG3 was an ISG15 transcriptional inhibitor.** (A) ChIP assay determined the enrichment of ISG15 promoter by anti-PEG3. (B) EMSA assay assessed the binding relationship between PEG3 protein and ISG15 promoter. (C) ISG15 promoter segmentations to construct dual-luciferase reporter and luciferase activity was detected. (D, E) Dual-luciferase reporter assay verified the binding site of PEG3 on ISG15 promoter. N=3. Mean ± SD. ****P*<0.001.

**Figure S3. PEG3 overexpression reduced cell viability and mitophagy levels.** (A) QPCR analysis evaluated the mRNA expression levels of PEG3 in transfections. (B) WB analysis determined the protein expression levels of PEG3, ISG15 and VCP in transfections, using β-actin as the reference. (C-D) CCK-8 measured the cell activity of TPC-1 and IHH-4 cells. (E) WB analysis determined the protein expression levels of mitophagy biomarkers of LC3, Parkin, and PINK1 in transfections, using β-actin as the reference. (F-G) The p-S65-Ub and mitochondrial Parkin level was assessed by WB. (H) The co-localization of LC3B and TOMM20 was determined by IF assay. N=3. Mean ± SD. ***P*<0.01, ****P*<0.001 *vs.* vector or TPC-1-vector. ^$$$^ P<0.001, *vs.* IHH-4-vector.
